# Supplementary material for: Expression Pattern of Tenascin-C, Matrilin-2, and Aggrecan in Diseases Affecting the Corneal Endothelium
Source: J Clin Med. 2022 Oct 11;11(20):5991. doi: 10.3390/jcm11205991 (PMC9604752; doi:10.3390/jcm11205991)
Supplement: Supplementary file 1 [file jcm-11-05991-s001.zip › SUPPLEMENTARY MATERIAL ONLINE_jcm-1866388.pdf]

SUPPLEMENTARY MATERIAL ONLINE

Expression pattern of tenascin-C, matrilin-2, and aggrecan in corneal endothelial pathological conditions

**Gréta Varkoly [1], Tibor G. Hortobágyi [2], Enikő Gebri [3], János Bencze [4], Tibor Hortobágyi [5,6,7,8,*,†]
and László Módis, Jr. [9,†]**

Table S1. The results of Kruskal-Wallis test

Table Analyzed	LAYER_MATRILLIN_CONTROL	Table Analyzed	LAYER_MATRILLIN_PBK	Table Analyzed	LAYER_MATRILIN_FD
Kruskal-Wallis test		*Kruskal-Wallis test*		*Kruskal-Wallis test*	
P value	<0,0001	P value	<0,0001	P value	0,1769
Exact or approximate P value?	Approximate	Exact or approximate P value?	Approximate	Exact or approximate P value?	Approximate
P value summary	****	P value summary	****	P value summary	ns
Do the medians vary signif. (P < 0.05)?	Yes	Do the medians vary signif. (P < 0.05)?	Yes	Do the medians vary signif. (P < 0.05)?	No
Number of groups	7	Number of groups	7	Number of groups	7
Kruskal-Wallis statistic	68,46	Kruskal-Wallis statistic	71,26	Kruskal-Wallis statistic	8,941
Data summary		*Data summary*		*Data summary*	
Number of treatments (columns)	7	Number of treatments (columns)	7	Number of treatments (columns)	7

Number of values (total)	77	Number of values (total)	132	Number of values (total)	61
Table Analyzed	LAYER_TENASCIN_CONTROL	Table Analyzed	LAYER_TENASCIN_PBK	Table Analyzed	LAYER_TENASCIN_FD
Kruskal-Wallis test		*Kruskal-Wallis test*		*Kruskal-Wallis test*	
P value	0,0016	P value	<0,0001	P value	<0,0001
Exact or approximate P value?	Approximate	Exact or approximate P value?	Approximate	Exact or approximate P value?	Approximate
P value summary	**	P value summary	****	P value summary	****
Do the medians vary signif. (P < 0.05)?	Yes	Do the medians vary signif. (P < 0.05)?	Yes	Do the medians vary signif. (P < 0.05)?	Yes
Number of groups	7	Number of groups	7	Number of groups	7
Kruskal-Wallis statistic	21,29	Kruskal-Wallis statistic	67,03	Kruskal-Wallis statistic	28,59
Data summary		*Data summary*		*Data summary*	
Number of treatments (columns)	7	Number of treatments (columns)	7	Number of treatments (columns)	7
Number of values (total)	69	Number of values (total)	131	Number of values (total)	53
Table Analyzed	LAYER_AGGRECAN_CONTROL	Table Analyzed	LAYER_AGGRECAN_PBK	Table Analyzed	LAYER_AGGRECAN_FD

Kruskal-Wallis test		*Kruskal-Wallis test*		*Kruskal-Wallis test*	
P value	<0,0001	P value	<0,0001	P value	0,002
Exact or approximate P value?	Approximate	Exact or approximate P value?	Approximate	Exact or approximate P value?	Approximate
P value summary	****	P value summary	****	P value summary	**
Do the medians vary signif. (P < 0.05)?	Yes	Do the medians vary signif. (P < 0.05)?	Yes	Do the medians vary signif. (P < 0.05)?	Yes
Number of groups	7	Number of groups	7	Number of groups	7
Kruskal-Wallis statistic	50,56	Kruskal-Wallis statistic	36,28	Kruskal-Wallis statistic	20,78
Data summary		*Data summary*		*Data summary*	
Number of treatments (columns)	7	Number of treatments (columns)	7	Number of treatments (columns)	7
Number of values (total)	69	Number of values (total)	131	Number of values (total)	54

Table S2. Comparison of tenascin-C expression in different corneal layers within the investigated groups

	Control	PBK	FECD
Epithelium vs Bowman's membrane	0.0217	0.0013	ns
Epithelium vs. Anterior stroma	ns	ns	ns
Epithelium vs. Middle stroma	0.0194	ns	ns
Epithelium vs. Posterior stroma	ns	ns	ns
Epithelium vs. Descemet's membrane	0.0033	<0.0001	0.0078
Epithelium vs. Endothelium	ns	ns	ns
Bowman's membrane vs. Anterior stroma	ns	ns	ns
Bowman's membrane vs. Middle stroma	ns	ns	ns
Bowman's membrane vs. Posterior stroma	ns	0.0005	ns
Bowman's membrane vs. Descemet's membrane	ns	ns	ns

Bowman's membrane vs. Endothelium	ns	ns	ns
Anterior stroma vs. Middle stroma	ns	ns	ns
Anterior stroma vs. Posterior stroma	ns	ns	ns
Anterior stroma vs. Descemet's membrane	ns	<0.0001	ns
Anterior stroma vs. Endothelium	ns	ns	ns
Middle stroma vs. Posterior stroma	ns	ns	ns
Middle stroma vs. Descemet's membrane	ns	<0.0001	ns
Middle stroma vs. Endothelium	ns	ns	ns
Posterior stroma vs. Descemet's membrane	ns	<0.0001	<0.0001
Posterior stroma vs. Endothelium	ns	0.0373	ns
Descemet's membrane vs. Endothelium	ns	0.0482	0.0187

PBK=pseudophakic bullous keratopathy, FECD=Fuchs' endothelial corneal dystrophy, ns=no significance. Red numbers indicate significant adjusted p values.

Table S3. Comparison of matrilin-2 expression in different corneal layers within the investigated groups

	Control	PBK	FECD
Epithelium vs Bowman's membrane	0.0012	<0.0001	ns
Epithelium vs. Anterior stroma	<0.0001	<0.0001	ns
Epithelium vs. Middle stroma	<0.0001	<0.0001	ns
Epithelium vs. Posterior stroma	<0.0001	<0.0001	ns
Epithelium vs. Descemet's membrane	<0.0001	<0.0001	ns
Epithelium vs. Endothelium	ns	0.0022	ns
Bowman's membrane vs. Anterior stroma	ns	ns	ns
Bowman's membrane vs. Middle stroma	ns	ns	ns
Bowman's membrane vs. Posterior stroma	ns	ns	ns
Bowman's membrane vs. Descemet's membrane	ns	ns	ns

Bowman's membrane vs. Endothelium	0.0002	ns	ns
Anterior stroma vs. Middle stroma	ns	ns	ns
Anterior stroma vs. Posterior stroma	ns	ns	ns
Anterior stroma vs. Descemet's membrane	ns	ns	ns
Anterior stroma vs. Endothelium	<0.0001	ns	ns
Middle stroma vs. Posterior stroma	ns	ns	ns
Middle stroma vs. Descemet's membrane	ns	ns	ns
Middle stroma vs. Endothelium	<0.0001	0.0121	ns
Posterior stroma vs. Descemet's membrane	ns	ns	ns
Posterior stroma vs. Endothelium	<0.0001	ns	ns
Descemet's membrane vs. Endothelium	<0.0001	0.0343	ns

PBK=pseudophakic bullous keratopathy, FECD=Fuchs' endothelial corneal dystrophy, ns=no significance. Red numbers indicate significant adjusted p values.

Table S4. Comparison of aggrecan expression in different corneal layers within the investigated groups

	Control	PBK	FECD
Epithelium vs Bowman's membrane	ns	ns	ns
Epithelium vs. Anterior stroma	<0.0001	ns	ns
Epithelium vs. Middle stroma	<0.0001	ns	ns
Epithelium vs. Posterior stroma	<0.0001	ns	ns
Epithelium vs. Descemet's membrane	<0.0001	<0.0001	ns
Epithelium vs. Endothelium	ns	ns	ns
Bowman's membrane vs. Anterior stroma	0.0442	ns	ns
Bowman's membrane vs. Middle stroma	0.0442	ns	ns
Bowman's membrane vs. Posterior stroma	0.0442	ns	ns
Bowman's membrane vs. Descemet's	0.0442	0.0004	0.0115

membrane			
Bowman's membrane vs. Endothelium	ns	ns	ns
Anterior stroma vs. Middle stroma	ns	ns	ns
Anterior stroma vs. Posterior stroma	ns	ns	ns
Anterior stroma vs. Descemet's membrane	ns	ns	ns
Anterior stroma vs. Endothelium	0.0079	ns	ns
Middle stroma vs. Posterior stroma	ns	ns	ns
Middle stroma vs. Descemet's membrane	ns	ns	ns
Middle stroma vs. Endothelium	0.0079	ns	ns
Posterior stroma vs. Descemet's membrane	ns	0.004	0.0364
Posterior stroma vs. Endothelium	0.0079	ns	ns
Descemet's membrane vs. Endothelium	0.0079	0.0009	ns

PBK=pseudophakic bullous keratopathy, FECD=Fuchs' endothelial corneal dystrophy, ns=no significance. Red numbers indicate significant adjusted p values.
