# Supplementary material for: Expression Pattern of Tenascin-C, Matrilin-2, and Aggrecan in Diseases Affecting the Corneal Endothelium
Source: J Clin Med. 2022 Oct 11;11(20):5991. doi: 10.3390/jcm11205991 (PMC9604752; doi:10.3390/jcm11205991)
Supplement: Supplementary file 1 [file jcm-11-05991-s001.zip › Table S1.pdf]

Table S1. The results of Kruskal-Wallis tes

Table Analyzed	**LAYER_MATRILLIN_CONTROL**	Table Analyzed	**LAYER_MATRILLIN_PBK**	Table Analyzed	**LAYER_MATRILIN_FD**
Kruskal-Wallis test		***Kruskal-Wallis test***		***Kruskal-Wallis test***	
P value	<0,0001	P value	<0,0001	P value	0,1769
Exact or approximate P value?	Approximate	Exact or approximate P value?	Approximate	Exact or approximate P value?	Approximate
P value summary	****	P value summary	****	P value summary	ns
Do the medians vary signif. (P < 0.05)?	Yes	Do the medians vary signif. (P < 0.05)?	Yes	Do the medians vary signif. (P < 0.05)?	No
Number of groups	7	Number of groups	7	Number of groups	7
Kruskal-Wallis statistic	68,46	Kruskal-Wallis statistic	71,26	Kruskal-Wallis statistic	8,941
Data summary		***Data summary***		***Data summary***	
Number of treatments (columns)	7	Number of treatments (columns)	7	Number of treatments (columns)	7
Number of values (total)	77	Number of values (total)	132	Number of values (total)	61
Table Analyzed	**LAYER_TENASCIN_CONTROL**	Table Analyzed	**LAYER_TENASCIN_PBK**	Table Analyzed	**LAYER_TENASCIN_FD**
Kruskal-Wallis test		***Kruskal-Wallis test***		***Kruskal-Wallis test***	
P value	0,0016	P value	<0,0001	P value	<0,0001
Exact or approximate P value?	Approximate	Exact or approximate P value?	Approximate	Exact or approximate P value?	Approximate
P value summary	**	P value summary	****	P value summary	****

Do the medians vary signif. (P < 0.05)?	Yes	Do the medians vary signif. (P < 0.05)?	Yes	Do the medians vary signif. (P < 0.05)?	Yes
Number of groups	7	Number of groups	7	Number of groups	7
Kruskal-Wallis statistic	21,29	Kruskal-Wallis statistic	67,03	Kruskal-Wallis statistic	28,59
Data summary		***Data summary***		***Data summary***	
Number of treatments (columns)	7	Number of treatments (columns)	7	Number of treatments (columns)	7
Number of values (total)	69	Number of values (total)	131	Number of values (total)	53
Table Analyzed	**LAYER_AGGRECAN_CONTROL**	Table Analyzed	**LAYER_AGGRECAN_PBK**	Table Analyzed	**LAYER_AGGRECAN_FD**
Kruskal-Wallis test		***Kruskal-Wallis test***		***Kruskal-Wallis test***	
P value	<0,0001	P value	<0,0001	P value	0,002
Exact or approximate P value?	Approximate	Exact or approximate P value?	Approximate	Exact or approximate P value?	Approximate
P value summary	****	P value summary	****	P value summary	**
Do the medians vary signif. (P < 0.05)?	Yes	Do the medians vary signif. (P < 0.05)?	Yes	Do the medians vary signif. (P < 0.05)?	Yes
Number of groups	7	Number of groups	7	Number of groups	7
Kruskal-Wallis statistic	50,56	Kruskal-Wallis statistic	36,28	Kruskal-Wallis statistic	20,78
Data summary		***Data summary***		***Data summary***	
Number of treatmen	7	Number of treatmen	7	Number of treatmen	7

ts (columns)		ts (columns)		ts (columns)	
Number of values (total)	69	Number of values (total)	131	Number of values (total)	54
