# Supplementary material for: Expression Pattern of Tenascin-C, Matrilin-2, and Aggrecan in Diseases Affecting the Corneal Endothelium
Source: J Clin Med. 2022 Oct 11;11(20):5991. doi: 10.3390/jcm11205991 (PMC9604752; doi:10.3390/jcm11205991)
Supplement: Supplementary file 1 [file jcm-11-05991-s001.zip › Table S3.pdf]

Table S3. Comparison of matrilin-2 expression in different corneal layers within the investigated groups

	Control	PBK	FECD
Epithelium vs Bowman's membrane	**0.0012**	**<0.0001**	ns
Epithelium vs. Anterior stroma	**<0.0001**	**<0.0001**	ns
Epithelium vs. Middle stroma	**<0.0001**	**<0.0001**	ns
Epithelium vs. Posterior stroma	**<0.0001**	**<0.0001**	ns
Epithelium vs. Descemet's membrane	**<0.0001**	**<0.0001**	ns
Epithelium vs. Endothelium	ns	**0.0022**	ns
Bowman's membrane vs. Anterior stroma	ns	ns	ns
Bowman's membrane vs. Middle stroma	ns	ns	ns
Bowman's membrane vs. Posterior stroma	ns	ns	ns
Bowman's membrane vs. Descemet's membrane	ns	ns	ns
Bowman's membrane vs. Endothelium	**0.0002**	ns	ns
Anterior stroma vs. Middle stroma	ns	ns	ns
Anterior stroma vs. Posterior stroma	ns	ns	ns
Anterior stroma vs. Descemet's membrane	ns	ns	ns
Anterior stroma vs. Endothelium	**<0.0001**	ns	ns
Middle stroma vs. Posterior stroma	ns	ns	ns

Middle stroma vs. Descemet's membrane	ns	ns	ns
Middle stroma vs. Endothelium	**<0.0001**	**0.0121**	ns
Posterior stroma vs. Descemet's membrane	ns	ns	ns
Posterior stroma vs. Endothelium	**<0.0001**	ns	ns
Descemet's membrane vs. Endothelium	**<0.0001**	**0.0343**	ns

PBK=pseudophakic bullous keratopathy, FECD=Fuchs' endothelial corneal dystrophy, ns=no significance. Red numbers indicate significant adjusted p values.
