# Supplementary material for: Expression Pattern of Tenascin-C, Matrilin-2, and Aggrecan in Diseases Affecting the Corneal Endothelium
Source: J Clin Med. 2022 Oct 11;11(20):5991. doi: 10.3390/jcm11205991 (PMC9604752; doi:10.3390/jcm11205991)
Supplement: Supplementary file 1 [file jcm-11-05991-s001.zip › Table S4.pdf]

Table S4. Comparison of aggrecan expression in different corneal layers within the investigated groups

	Control	PBK	FECD
Epithelium vs Bowman's membrane	ns	ns	ns
Epithelium vs. Anterior stroma	**<0.0001**	ns	ns
Epithelium vs. Middle stroma	**<0.0001**	ns	ns
Epithelium vs. Posterior stroma	**<0.0001**	ns	ns
Epithelium vs. Descemet's membrane	**<0.0001**	**<0.0001**	ns
Epithelium vs. Endothelium	ns	ns	ns
Bowman's membrane vs. Anterior stroma	**0.0442**	ns	ns
Bowman's membrane vs. Middle stroma	**0.0442**	ns	ns
Bowman's membrane vs. Posterior stroma	**0.0442**	ns	ns
Bowman's membrane vs. Descemet's membrane	**0.0442**	**0.0004**	**0.0115**
Bowman's membrane vs. Endothelium	ns	ns	ns
Anterior stroma vs. Middle stroma	ns	ns	ns
Anterior stroma vs. Posterior stroma	ns	ns	ns
Anterior stroma vs. Descemet's membrane	ns	ns	ns
Anterior stroma vs. Endothelium	**0.0079**	ns	ns
Middle stroma vs. Posterior stroma	ns	ns	ns

Middle stroma vs. Descemet's membrane	ns	ns	ns
Middle stroma vs. Endothelium	**0.0079**	ns	ns
Posterior stroma vs. Descemet's membrane	ns	**0.004**	**0.0364**
Posterior stroma vs. Endothelium	**0.0079**	ns	ns
Descemet's membrane vs. Endothelium	**0.0079**	**0.0009**	ns

PBK=pseudophakic bullous keratopathy, FECD=Fuchs' endothelial corneal dystrophy, ns=no significance. Red numbers indicate significant adjusted p values.
